# Genome-wide transcriptomic analysis of response to low temperature reveals candidate genes determining divergent cold-sensitivity of maize inbred lines

**DOI:** 10.1007/s11103-014-0187-8

**Published:** 2014-03-13

**Authors:** Alicja Sobkowiak, Maciej Jończyk, Emilia Jarochowska, Przemysław Biecek, Joanna Trzcinska-Danielewicz, Jörg Leipner, Jan Fronk, Paweł Sowiński

**Affiliations:** 1 Plant Biochemistry and Physiology Department, Plant Breeding and Acclimatization Institute - National Research Institute, Radzików, 05-870 Błonie, Poland; 2 Department of Plant Molecular Ecophysiology, Faculty of Biology, Institute of Plant Experimental Biology and Biotechnology, University of Warsaw, 02-096 Warszawa, Miecznikowa 1, Poland; 3 Faculty of Mathematics, Informatics and Mechanics, Institute of Applied Mathematics and Mechanics, University of Warsaw, 02-097 Warszawa, Banacha 2, Poland; 4 Department of Molecular Biology, Faculty of Biology, Institute of Biochemistry, University of Warsaw, 02-096 Warszawa, Miecznikowa 1, Poland; 5 Department of Environmental Systems Science, ETH Zürich, Universitätstrasse 2, 8092 Zurich, Switzerland; 6 Present Address: GeoZentrum Nordbayern, Fachgruppe Paläoumwelt, Universität Erlangen-Nürnberg, Loewenichstr. 28, 91054 Erlangen, Germany

**Keywords:** Gene ontology, Low temperature, qRT PCR, QTL, Transcriptomics, *Zea mays*

## Abstract

Maize, despite being thermophyllic due to its tropical origin, demonstrates high intraspecific diversity in cold-tolerance. To search for molecular mechanisms of this diversity, transcriptomic response to cold was studied in two inbred lines of contrasting cold-tolerance. Microarray analysis was followed by extensive statistical elaboration of data, literature data mining, and gene ontology-based classification. The lines used had been bred earlier specifically for determination of QTLs for cold-performance of photosynthesis. This allowed direct comparison of present transcriptomic data with the earlier QTL mapping results. Cold-treated (14 h at 8/6 °C) maize seedlings of cold-tolerant ETH-DH7 and cold-sensitive ETH-DL3 lines at V3 stage showed strong, consistent response of the third leaf transcriptome: several thousand probes showed similar, statistically significant change in both lines, while only tens responded differently in the two lines. The most striking difference between the responses of the two lines to cold was the induction of expression of ca. twenty genes encoding membrane/cell wall proteins exclusively in the cold-tolerant ETH-DH7 line. The common response comprised mainly repression of numerous genes related to photosynthesis and induction of genes related to basic biological activity: transcription, regulation of gene expression, protein phosphorylation, cell wall organization. Among the genes showing differential response, several were close to the QTL regions identified in earlier studies with the same inbred lines and associated with biometrical, physiological or biochemical parameters. These transcripts, including two apparently non-protein-coding ones, are particularly attractive candidates for future studies on mechanisms determining divergent cold-tolerance of inbred maize lines.

**Electronic supplementary material:**

The online version of this article (doi:10.1007/s11103-014-0187-8) contains supplementary material, which is available to authorized users.

## Introduction

The sensitivity of maize to low temperature is intensively studied for two main reasons. Firstly, maize is an important crop whose successful cultivation in temperate climate was primarily limited by its photoperiodic and thermal requirements. While modern varieties no longer show a great sensitivity to the photoperiod, low temperature remains a significant obstacle, especially at high latitudes. Secondly, there is a purely theoretical aspect. Maize, a C_4_ plant, has been a favorite object of manifold basic research in physiology, biochemistry, molecular biology and genetics. It is now becoming a model plant species for all studies where the cold-hardy C_3_ plant model *Arabidopsis thaliana* has little to offer.

The agricultural praxis is to sow maize when the soil temperature is above 8 °C. Seedling development in early spring is limited by the ability of a given material (line, hybrid, etc.) to quickly develop the first leaf at a low temperature and begin fully autotrophic growth (Sowiński et al. 2005). Also periods of low temperature occasionally encountered in May and June in many temperate regions are a significant threat for maize cultivation (Adamczyk and Królikowski 1998). Suboptimal temperatures in the range of 10–15 °C decrease the capacity for biomass production and lead to growth retardation, while even lower temperatures (2–8 °C) may cause irreversible damage and loss of plants (reviewed in: Greaves 1996; Foyer et al. 2002; Marocco et al. 2005; Leipner and Stamp 2009). Despite the fact that maize is generally sensitive to low temperatures, there is considerable variation within the maize germplasm in the extent of the cold sensitivity (Greaves 1996).

Studies on the mechanisms of the maize cold sensitivity have focused mostly on the functioning of photosynthesis at suboptimal temperatures, in particular retardation of chloroplast development (Nie et al. 1995), inhibition of photosynthetic enzymes (Kingston-Smith and Foyer 2000), lowering of photosynthetic quantum yield (Fryer et al. 1995), alterations of the pigment composition (Haldimann 1998), and the role of antioxidant systems (Leipner et al. 1999; Iannelli et al. 1999; Kocsy et al. 1996). Some role has also been postulated for feedback inhibition of photosynthesis by product accumulation due to an impediment of assimilates’ export from photosynthetic cells to the vascular parenchyma by partial obstruction of plasmodesmata (Bilska and Sowiński 2010). Furthermore, compromised root functioning leading to water and nutrient limitation has been proposed to contribute to the chilling sensitivity (Richner et al. 1996, 1997).

For many years attempts have been undertaken to establish the molecular basis of the maize chilling sensitivity. Most of these studies were focused on the effect of severe cold stress (<8 °C). Using classical methods of molecular genetics, several genes taking part in the maize response to cold have been identified, mostly related to carbohydrate and secondary metabolism (Marocco et al. 2005). The use of suppression subtractive hybridization allowed a larger-scale study which identified several genes related to photosynthesis, sugar metabolism and signal transduction (Nguyen et al. 2009; Zhang et al. 2009). On the other hand, in a microarray study of the maize response to moderately low temperatures (>10 °C) genes related to circadian regulation and cell wall functioning, but not to photosynthesis, were found to be affected (Trzcinska-Danielewicz et al. 2009). Each of those studies used only a single inbred line, which made impossible the identification of the genetic basis of the contrasting cold tolerance/sensitivity among maize genotypes.

Until now attempts to explain the genetic basis of the differences among maize materials in respect to their cold-tolerance have been limited to quantitative trait locus (QTL) analyses. Several studies have been carried out, leading to the identification of a number of QTLs of potential relevance for growth at low temperature. These studies allowed also identifying traits underlying the cold-induced reduction in seedling growth. In a Lo964 × Lo1016 population grown under suboptimal temperature, QTLs were found for shoot dry weight which colocalized with QTLs for leaf area, leaf greenness and/or PSII operating efficiency (Hund et al. 2004). Presterl et al. (2007) identified several QTLs for shoot fresh weight at early growth stages under contrasting temperature conditions in the field; several of those QTLs were associated with leaf chlorosis.

A comprehensive set of QTL studies have been performed with a QTL mapping population developed specifically for this purpose. Inbred lines with a contrasting cold tolerance of photosynthesis were obtained by divergent selection from a Swiss dent maize breeding population using the PSII operating efficiency (as defined in Genty et al. 1989) as the selection criterion (Fracheboud et al. 1999). This parameter was measured at low temperature and low light intensity in maize seedlings that developed at moderately low temperatures. Two lines denoted ETH-DH7 (cold-tolerant) and ETH-DL3 (cold-sensitive) were used to generate a QTL mapping population (Fracheboud et al. 2004) which was then used in several QTL studies performed under controlled and field conditions. The major QTLs for cold tolerance of photosynthesis (determined by chlorophyll fluorescence and gas-exchange analysis) were stable across cold environments (growth chamber and field). Furthermore, they were often associated with the leaf greenness (chlorophyll content), and in some cases with the seedling shoot dry weight or leaf biometric characteristics (leaf length or specific leaf area) (Fracheboud et al. 2004; Jompuk et al. 2005; Hund et al. 2005). No or only poor association was found between the traits for cold tolerance of photosynthesis and the extractable activity of photosynthetic enzymes, e.g., malate dehydrogenase, or the amount of antioxidants, e.g., α-tocopherol (Leipner and Mayer 2008). The authors of those studies discussed potential candidate genes related to the trait, e.g., *hcf106*, *psaH* and *cab*-*m7* associated with the photosynthetic machinery, but could not verify them (for summary see Leipner 2009). One should bear in mind, however, that the resolution of QTL mapping is rather low and numerous genes can be found in proximity of a given marker. For that reason, additional information is required to choose from among such adjacent genes the ones that actually affect the trait in question.

Another approach for identification of genes related to particular traits, in some aspects complementary to QTL mapping, relies on high-throughput analyses of gene expression based on microarray hybridization or deep sequencing of the transcriptome. The large bodies of data obtained by these methods require sophisticated bioinformatic and statistical elaboration to be meaningful, but when properly analyzed they deliver an unprecedented wealth of information on gene expression patterns, mutual relationships among individual genes and gene groups, and also responses to diverse stimuli, including stress conditions.

In order to better understand the molecular mechanisms of the cold response of maize seedlings and to identify potential candidate genes that could explain the differences in the cold tolerance between different genotypes, we used microarray analysis to compare the transcriptomic responses to cold stress of two maize inbred lines with a contrasting cold-tolerance of photosynthesis. We used the two lines of divergent cold-sensitivity, ETH-DH7 and ETH-DL3, bred for the QTL analyses mentioned above, with the aim to conduct a comparative analysis of these results and the previously identified QTLs.

## Materials and methods

### Plant material

Two maize (*Zea mays* L.) inbred lines of dent type differing in the level of cold-tolerance, ETH-DH7 (cold-tolerant) and ETH-DL3 (cold-sensitive), were used. The lines had been produced at the Swiss Federal Institute of Technology Zurich (ETH Zurich) specifically to be used for mapping of QTLs associated with the cold tolerance of photosynthesis (Fracheboud et al. 2004). Kernels were germinated for 3 days in darkness at 25 °C. Seedlings were transferred to pots containing Knop’s solution and were grown in a growth chamber (photoperiod: 14/10 h day/night, light irradiance: 250 μmol quanta m^−2^ s^−1^, temperature: 24/22 °C and relative humidity 60/80 %) When the third leaf was fully developed (i.e., ligular region of the leaf was formed), at the end of the light period half of the plants were transferred to 8/6 °C (day/night) without changing the other conditions; the other half were grown in the same conditions as before (control plants). After 14 h, the middle part of the third leaf was collected from three plants for each treatment and genotype combination, pooled, flash-frozen in liquid nitrogen and stored at −80 °C until RNA isolation. Three fully independent consecutive biological experiments were performed.

### Chlorophyll fluorescence determinations

Maximum quantum efficiency of PSII primary photochemistry (Fv/Fm) and PSII operating quantum efficiency (Φ_PSII_) were measured with a fluorometer (PAM 200, H. Walz, Germany) in control plants and in plants subjected to low temperature (8/6 °C day/night) for 14, 38 or 110 h (i.e., 4 h after beginning of the light phase of photoperiod). For the measurement of Fv/Fm, the saturating one-second light flash intensity was about 3,500 μmol quanta m^−2^ s^−1^. Before measurements of Fv/Fm, the plants were dark-adapted for 30–60 min at 24 °C. For the measurement of Φ_PSII_ at 24 °C, leaves were exposed to eight 2-min periods of light of 200 μmol quanta m^−2^ s^−1^ intensity, each period terminated with a saturating 1-s light flash of ca. 3,500 μmol quanta m^−2^ s^−1^ intensity. Experiments were repeated three times using three plants per line per experimental variant. Data were subjected to two-way ANOVA with the use of PROC GLM procedure (SAS 9.1.3; SAS Institute, Cary, NC). Effects of line, variant and their interaction were considered as fixed effects while Fv/Fm and ΦPSII were considered as responding variables. Analyzes were performed at 0.05 significance level.

### Microarray description

Microarrays designed and produced by the Maize Oligonucleotide Array Project, University of Arizona, Tucson, USA (Gardiner et al. 2005) were used. They comprised 46,128 probes, mainly 70-mer, but also some 40- and 50-mer, as well as positive, negative and print controls.

### RNA isolation, purification, amplification and hybridization

RNA isolation, amplification, labeling, and hybridization followed the procedure described earlier (Jończyk et al. 2011). Comparative pairwise hybridizations were performed for all six combinations of line [ETH-DH7 (cold-tolerant) or ETH-DL3 (cold-sensitive)] and temperature (control or cold-treated). Separate sets of hybridizations were done for each of the three biological replications (see “Plant material” above). To minimize the effect of potential dye-bias, labeling was repeated with a dye (cy3, cy5) swap. Thus, a total of 36 hybridizations were done. Slide scanning was done with GenePix 4000B (Molecular Devices) and feature extraction with GenePix Pro 3.0 software.

### Data normalization and statistical analysis

Results of microarray scanning were normalized (lowess), controlled for quality (Principal Component Analysis, PCA), and statistically analyzed to identify genes responding to cold-treatment in the two maize lines. The experiment was modeled as an effect of the interaction in a two-way ANOVA model of the form: intensity = line + treatment + line:treatment. The maize inbred line (ETH-DH7/ETH-DL3) and treatment (control/cold) were considered as fixed effects while logarithm of normalized fluorescence intensity was used as the responding variable. This approach allowed identifying genes responding similarly in the two lines and those showing statistically different response between lines.

In order to normalize fluorescence intensity we performed a standard microarray background correction and normalization with the lowess method. After that, in order to remove a possible dye-bias we considered a model with dye (cy5/cy3) as the fixed effect with biological replicate and gene as confounding random effects. The estimated dye-effect was then subtracted from the fluorescence values. Note that the dye effect should in principle be minimized in this experiment since each condition was replicated three times with either dye (dye-swap), but by including the dye effect in the normalization phase we improved the estimation of the variance in the following two-way ANOVA model with interaction (Bates 2005).

The *p* values were calculated with the likelihood ratio test (LTR) between two-way models with and without interaction. These *p* values were adjusted for multiple comparisons with the Benjamini–Hochberg correction (Benjamini and Hochberg 1995) which controls for False Discovery Rate (FDR).

The R software was used to fit all ANOVA models, to perform statistical tests for interactions, and to calculate the adjusted *p* values (R Development Core Team 2008). The significance level for the interaction between line effect and treatment effect was set to either 0.05 or 0.1. Additionally, a set of genes showing a common response to low temperature in both lines was constructed comprising genes showing an effect of temperature (*p* < 0.05) minus those showing an effect of line (*p* < 0.05) and of interaction of line and temperature effects (*p* < 0.05).

Microarray experiments were described in compliance with MIAME (Minimum Information About Microarray Experiment; Brazma et al. 2001) guidelines. Raw microarray data have been deposited at the ArrayExpress database (www.ebi.ac.uk/arrayexpress) under experiment accession number E-MTAB-1315.

### Global and detailed data analysis

Global analysis was done with the use of the gene ontology hierarchical system and enrichment analysis. GO annotations were assigned to probes as described before (Jończyk et al. 2011). As a result, 57 % of the microarray probes obtained GO annotations. Over-represented GO categories were detected with the Ontologizer program (Grossmann et al. 2007; http://compbio.charite.de/contao/index.php/cmdlineOntologizer.html) using the “−i” option to ignore in the calculation genes without a GO annotation. The “Parent–Child Union” method was used which takes into account the hierarchical structure of the GO system, and the FDR correction (Benjamini and Hochberg 1995) was set at 0.05. Only non-redundant sequences were considered, i.e., when several probes matched a single gene, it was counted only once. The GO graphs were drawn with the GraphViz software (www.graphviz.org) integrated in the Ontologizer program.

Detailed analysis was based on literature data mining with Pathway Studio 9.0 (Elsevier). To use this program the identifiers of the genes of interest were converted to entrez ids accepted by the software, as described earlier (Jończyk et al. 2011). With this approach ca. 70 % of the microarray probes could be subjected to further pathway analysis of gene interactions. For each gene showing a different pattern of response to low temperature in the two lines, additional annotations were assigned from the Maize Genome Sequencing Project website (http://www.maizesequence.org) and the InterPro database (http://www.ebi.ac.uk/interpro).

### Quantitative real-time PCR

Quantitative real-time PCR primers were designed for 28 sequences including 23 sequences representing transcripts showing differential response (defined above) to low temperature in the two maize inbred lines. As reference, glyceraldehyde 3-phosphate dehydrogenase gene (*GAPDH*) was used because it showed constant expression in all experimental variants. Primers (Table S1, Online Resource 1) were designed using the open access software from PREMIER Biosoft International (NetPrimer: www.premierbiosoft.com/netprimer) and Invitrogen (OligoPerfect Designer: http://tools.lifetechnologies.com/content.cfm?pageid=9716). One of the primers from each pair was designed to hybridize to a sequence corresponding to a fragment of the microarray probe sequence.

The plant material for qRT PCR analyses was derived from the same three biological experiments as for microarray analyses, but RNA was isolated (as described above) from separate plants. cDNA synthesis was performed with the SuperScript First Strand Synthesis System for RT-PCR kit (Invitrogen) according to the manufacturer’s protocol. Correct product size generated by each pair of primers was confirmed by PCR and agarose gel (2 %) electrophoresis. Real-time PCR was carried out in a MyiQ2 (Bio-Rad) thermocycler in 20-μl reaction volume using SYBR Green JumpStart Taq Ready Mix (Sigma) with primers at 400 nM each and 0.1 μl of cDNA from the reverse-transcription reaction. On each plate three technical replicates were performed for each sample and three for no-template control. The data were analyzed with iQ5 Optical System Software (Bio-Rad). The standard amplification protocol for qRT-PCR has been described elsewhere (Trzcinska-Danielewicz et al. 2009).

The Ct value was determined by the iQ5 software for all the samples and the mean values were used in the formula ΔΔCt = [(Ct_GOI K_ − Ct_HKG K_) − (Ct_GOI C_ − Ct_HKG C_)], where Ct_GOI_ and Ct_HKG_ are the threshold cycles of the gene of interest and the house-keeping reference gene (Dussault and Pouliot 2006), respectively (K—control sample, C—sample obtained from cold-treated plants). To confirm the linearity of the assay, the amplification efficiencies of the target and the reference genes were determined from a dilution series of sample cDNA and were approximately equal.

## Results

**Fig. 1:**
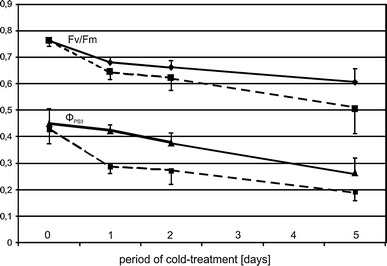
Basic parameters of photosynthetic apparatus efficiency in cold-treated maize. Maximal quantum yield of PS II, Fv/Fm (*upper lines*), and effective quantum yield of PS II, Φ_PSII_ (lower lines), of the 3rd leaf of ETH-DH7 (*solid*) and ETH-DL3 (*dashed*) plants grown at 24/22 °C (control, day 0) and plants treated with 8/6 °C for 14 h (night + 4 h of light), 28 h (two full photoperiods + 4 h of light) or for 124 h (five full photoperiods + 4 h of light). Data are means for 9 plants ± SD. For Fv/Fm comparisons between inbred lines, temperatures, and interaction of line and temperature effects the *p* values were 0.0001, 0.0001 and 0.0445, respectively; for Φ_PSII_ comparisons the respective *p* values were 0.0001, 0.0001 and 0.0211

To assess the physiological response of the maize seedlings to the low temperature conditions, the cold-response of F_v_/F_m_ and Φ_PSII_ was evaluated in both inbred lines. Under optimum growth conditions, there was no difference of Fv/Fm or Φ_PSII_ between the two inbred lines investigated (Fig. 1). However, already the shortest cold-treatment analyzed (14 h) caused a substantial reduction of both parameters, which became progressively more pronounced with the duration of the treatment. At all time points during the cold treatment the cold-sensitive ETH-DL3 line was more strongly affected than ETH-DH7. Thus, the sensitivity of the photosynthetic apparatus to low temperature showed the expected differences between the two lines. Moreover, the results indicated that a 14-h long cold treatment was sufficient to induce a substantial and genotype-specific physiological response. Having verified the suitability of the experimental regime employed we analyzed the effects of low temperature on the transcriptome of both lines. Here, only the shortest (14 h) treatment was evaluated in order to focus on the more direct molecular effects of cooling rather than secondary (acclimatory) effects.

The two lines, derived from a common gene pool, showed virtually identical gene expression patterns under control growth conditions (not shown, but see raw microarray data at ArrayExpress, acc. no. E-MTAB-1315). The transcriptome of both inbred lines responded strongly to the cold treatment. As many as 7,972 (17 %) probes showed a statistically significant change in expression in either line. For redundant probes (several probes corresponding to a single transcript), all but the most statistically significant one were removed from the set if the probes showed a consistent pattern of expression, or all were removed if they behaved inconsistently. We then set an additional criterion that the response must be at least 2.8-fold (|log_2_[(expression level in the cold):(expression level in control conditions)]| ≥ 1.5). As a result, we identified 2,449 cold-responsive genes behaving in a similar manner in both lines; of those, 1,059 showed repression and 1,390 induction of expression (Table S2, Online Resource 2). A statistically significantly different mode of cold-response between the two lines was found for 66 genes (Table S3, Online Resource 3). As explained earlier, the genes represented by redundant probes showing an inconsistent pattern of response were excluded from further considerations; such probes may, nevertheless, be of some interest, hence we list them in supplementary materials (Table S4, Online Resource 4).

### Common response: detailed analysis

**Fig. 2:**
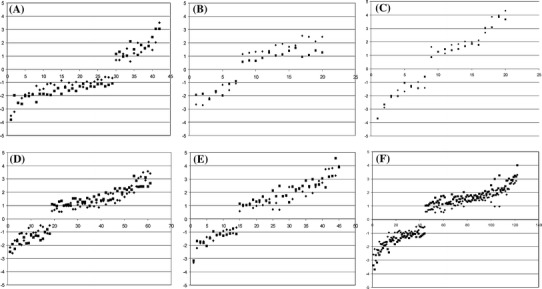
Major groups of genes responding to cold treatment in a similar manner in both maize lines. Change of transcript level [shown as log_2_(cold/control)] in leaves of two maize inbred lines (*filled diamond*—ETH-DH7, *filled square*—ETH-DL3) treated with cold. Arbitrary functional groups: **a** chloroplast functioning, **b** antioxidant systems, **c** circadian clock, **d** carbohydrate metabolism, **e** stress response, **f** transcription factors. Abscissa—gene number as listed in Table S5

Among the genes showing a similar response to low temperature in both lines several fairly numerous functional groups seemed of particular relevance (Fig. 2, Table S5—Online Resource 5). Consistent with the physiological data showing a cold-induced reduction of photosynthetic efficiency (Fig. 1, Table S5—Online Resource 5), chloroplast-related genes were preferentially repressed by low temperature (29 out of the 42 responding, Fig. 2A, Table S5—Online Resource 5). Such a massive change of the photosynthetic apparatus could be expected to affect the redox balance, however, that was apparently not the case since only a few genes encoding antioxidant enzymes were induced; most of them were peroxidases and glutathione transferases and showed only a moderate induction (Fig. 2B, Table S5—Online Resource 5).

One of the strongest responses to cold was shown by several putative components of the circadian clock, orthologs of *A. thaliana LHY, TOC1, Gigantea* and *PRR7* (Fig. 2C, Table S5—Online Resource 5), suggesting a strong disturbance of the circadian rhythm. Since the circadian clock and photoperiod regulate virtually all aspects of plant physiology (Webb 2003), we looked for other possible clock components among the genes responding to the cold treatment. The database was queried using known *A. thaliana* circadian clock genes and candidate maize clock genes found earlier (Jończyk et al. 2011). In that manner additional cold-modulated genes potentially involved in clock functioning were found, such as orthologs of *A. thaliana* *ELF4* and *LKP2*, rice circadian clock gene *PRR95*, and maize transcription factor-encoding genes *LOC100272621, NAC1* and *COL9* (Table S5—Online Resource 5).

Genes related to carbohydrate metabolism were mostly induced (42 out of the 61 responding to cold; Fig. 2D, Table S5—Online Resource 5). Among those genes, nine encoding enzymes of the photosynthetic dark-reaction were induced and three repressed, whilst six genes related to starch synthesis were repressed and only two were induced. These changes indicate substantial modification of primary carbon metabolism in both lines in the cold.

In addition to the above highly specific groups of cold-responsive genes three more-general ones are of particular interest. From the group of 45 stress-related genes, 31 were induced (Fig. 2E, Table S5—Online Resource 5). These genes are related to a large repertoire of stress factors, mostly biotic, but also abiotic ones, such as mechanical stress and wounding, heat, oxidative and aluminum stresses. Most common among the aforementioned genes were those previously found to be related to dehydration and response to drought, including orthologs of *A. thaliana* *DREB1A* and *CBF1*, known also for their activation at low temperature (Qin et al. 2004). Genes related to hormones (both growth stimulators and repressors) showed both induction (49) and repression (14) and comprised factors directly engaged in hormone metabolism as well as hormone-responsive genes (Table S5—Online Resource 5). Such a widespread modulation of expression of stress- and hormone-related genes points to profound physiological changes following a relatively short exposure to low temperature, and also indicates that at least part of the response is not specific to this particular stress. By far the most numerous was the group of transcription factor genes (Fig. 2F, Table S5—Online Resource 5). Here, induction slightly dominated over repression (77 vs. 45). Their modulation heralds massive changes of expression of effector genes, some visible already at the time of our analysis, but some likely to be part of delayed response. Taken together, these data show that despite their different cold-sensitivity the both lines reacted strongly to low temperature.

### Common response: global analysis

A gene-by-gene analysis such as that presented in the preceding paragraph, although informative, is—by its nature—rather subjective and of limited scope. To study objectively the relations among all the genes showing similar responses to low temperature in both lines, a global analysis was performed for over-represented (enriched) gene ontology categories.

A vast majority of the genes repressed in response to the cold treatment were distributed evenly among numerous GO categories, possibly reflecting a nonspecific general down-regulation of the plant’s activity. However, a small set of the repressed genes stood out; they belonged to four functionally related over-represented lowest-rank GO categories (only the lowest-rank categories are discussed, as they are the most informative). In the “Biological Process” class (Fig. S1A—Online Resource 6) these were GO:0006778 (porphyrin-containing compound metabolic process) and GO:0009765 (photosynthesis, light harvesting), in the “Molecular Function” class (Fig. S1B—Online Resource 7) GO:0016168 (chlorophyll binding), and in the “Cellular Component” class (Fig. S1C—Online Resource 8) GO:0009783 (photosystem II antenna complex). These findings, free from subjectivity, are in full support of the above detailed analysis and confirm the repression of genes related to the light phase of photosynthesis as a specific response to cold treatment common to the two lines.

In contrast to the repressed genes, those induced by cold stress seemed to reflect a highly specific response and mostly belonged to a small subset of categories. As many as ten over-represented GO categories were found in the “Biological Process” class (Fig. S2A—Online Resource 9). Among them, six were related to basic biological activity, like GO:2000112 (regulation of cellular macromolecule biosynthetic process), GO:0006468 (protein phosphorylation), GO:0006351 (transcription, DNA-dependent), GO:0010468 (regulation of gene expression), GO:0071554 (cell wall organization or biogenesis) and GO:0071702 (organic substance transport). The two latter correspond to enriched category GO:0000145 (exocyst) of “Cellular Component” class (Tab. S2B—Online Resource 10). Two GO categories, both related to fundamental processes, were over-represented in the “Molecular Function” class (Fig. S2C—Online Resource 11): GO:0003824 (catalytic activity) and GO:0003677 (DNA binding). This highly specific induction of genes likely indicates profound remodeling of maize leaf metabolism in response to low temperature, consistent with the conclusions based on the detailed analysis above.

### Differential response

**Fig. 3:**
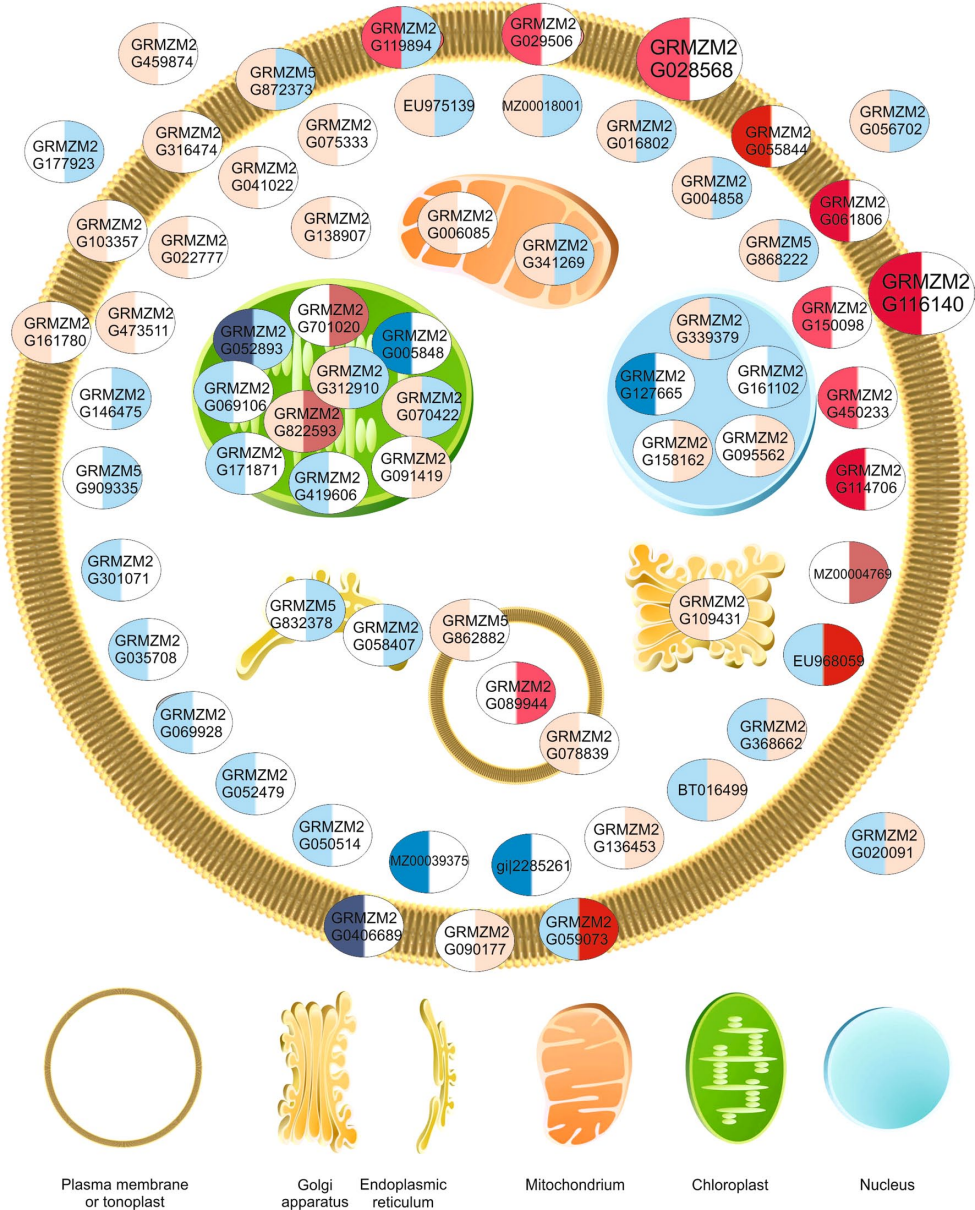
Predicted cellular localization of products of genes responding differentially to cold treatment in the two maize lines. Genes showing statistically significant (*p* < 0.1) interaction of inbred line and temperature effects for two maize inbred lines were analyzed. The localization of proteins was assigned basing on GO annotation (Cellular Component) or InterPro domain description. In each oval the left-hand half represents the response of the cold-tolerant ETH-DH7 line and the right-hand half—the response of the cold-sensitive ETH-DL3 line. Two larger ovals depict proteins related to both cell membrane and cell wall. *Colors* indicate the cold/control ratio (induction of expression—*red*, repression—*blue*, no change—*white*) and their intensity the magnitude of change. Detailed data on the proteins identified by ensembl gene IDs are presented in Table S3. The figure was drawn using the Pathway Studio v. 9.0 (Elsevier)

Besides the numerous genes showing a similar response to cold treatment in both lines, some genes responded significantly differently in these lines. To identify them, the interaction of inbred line and temperature effects was analyzed. The number of such genes was 26 or 66 when the cutoff *p* value for the interaction was set at 0.05 or 0.1, respectively. For further considerations the less restrictive cutoff value was used (Fig. 3; Table S3—Online Resource 3). One should note that “significantly different” response is a general term reflecting in fact any of three fundamentally different situations: a response of the same nature (that is, induction or repression) in both lines, but of statistically significantly different magnitude; a response (induction or repression) in one line and no response in the other; and a contrasting response, i.e., induction in one and repression in the other. These three possibilities can easily be discerned from the color-coded data in Fig. 3. We performed a detailed analysis of all 66 genes responding differently to low temperature in the two lines (Table S3—Online Resource 3). That analysis was based on literature data mining performed with Pathway Studio 9.0 supplemented with functional analysis of annotations from the Maize Genome Sequencing Project and Gramene databases and analysis of domains, repeats and sequence motifs using the InterPro system. Several of those genes were related to carbohydrate and amino acid metabolism, signal transduction pathways, and redox potential homeostasis. However, the most numerous were genes encoding proteins with a putative localization in membranes (including ER and tonoplast) or the cell wall, such as diverse transporters, ATPases, receptors and other proteins with transmembrane domains. Most of those genes showed a stronger induction of expression in the cold-tolerant ETH-DH7 line than in ETH-DL3, in which, actually, some of the genes did not respond at all or even were repressed. There was no obvious preference for any subcellular localization of encoded proteins among the less numerous genes showing a stronger induction in ETH-DL3.

### Quantitative real time RT-PCR

The microarray data were verified by quantitative RT-PCR. To this end, 28 genes (23 of which showed a differential response) were selected, representing different signal strength, from weak, close to the negative control level, up to the saturation level. Primers used are shown in Table S1. The *GAPDH* transcript was used as a reference with a steady level of expression. The results of the qRT PCR analysis are shown in Table S6 (Online Resource 12) together with corresponding microarray data. The correlation coefficients between the data obtained with these two methods are 0.98 and 0.95 for ETH-DH7 and ETH-DL3, respectively. The cold/control expression ratios calculated from the qRT-PCR results were also very close to those derived from the microarray data. Thus, the microarray results of this study faithfully represent the true abundances of individual transcripts.

## Discussion

Changes of gene expression patterns in response to cold stress were studied in leaves of the cold-tolerant maize ETH-DH7 line and the cold-sensitive ETH-DL3 (Fracheboud et al. 2004). By measuring two basic chlorophyll fluorescence parameters, Fv/Fm and Φ_PSII_, we verified that the two lines indeed showed a markedly different physiological response to the cold stress under the conditions used in the present study. Earlier studies using a much longer mild cold stress found similar differences in diverse chlorophyll fluorescence parameters, including Fv/Fm and Φ_PSII_ (Fracheboud et al. 2004).

Despite being of different cold sensitivity, the both lines showed a remarkably conserved transcriptional response to the cold treatment: ca. 2,500 genes showed a response common to both lines, and only 66 a different response. The genes from the first group are likely responsible for the general response of maize to the cold, while those from the second group represent the differential responses of the two inbred lines and could be associated with the genetic difference in the cold tolerance between them.

### Common response

Although the both lines studied had originally been selected for a maximally different efficiency of the photosynthetic apparatus at low temperature, they showed a surprisingly similar transcriptional response, i.e., repression of many genes related to chloroplasts, highlighted both in detailed and global analysis. This is likely due to the common genetic origin of the two lines, namely from a dent maize breeding population, and to the fact that the inbred lines had been selected for a contrasting photosynthetic efficiency of leaves that developed under low temperature rather than for a direct temperature response (Fracheboud et al. 2004). Most of the cold-repressed genes coded for light harvesting chlorophyll a/b binding (LHCB/CAB) proteins, which is partially in line with earlier data that showed that protein levels of the photosynthetic machinery were reduced in plants grown at low temperature, mostly proteins of the PSII reaction center and, to a lower extent, LHCII proteins (Nie and Baker 1991). Interestingly, we also observed a strongly disturbed expression of genes related to the circadian clock responsible for the morning induction of the LHCB/CAB genes, shown to be under temperature control in *A. thaliana* (McClung and Gutiérez 2010). Thus, one might hypothesize that the repression of the LHCB/CAB genes in maize was a consequence of the loss of the circadian expression rhythm at severe cold rather than a direct result of oxidative stress. A disruption of the diurnal rhythm in maize treated with moderate chilling (14 °C) was suggested earlier (Trzcinska-Danielewicz et al. 2009), but it involved just a few components of the circadian clock without a pronounced repression of genes related to chloroplasts. Conversely, up-regulation of several genes related to chloroplast function was found in that study, including those associated with transcription, RNA processing and protein transport through the chloroplast membrane, suggesting acclimation to the suboptimal temperature. This illustrates one of the numerous differences between the effects of moderate (Trzcinska-Danielewicz et al. 2009) and severe (this study) cold on gene expression in maize. Low temperature was found to affect circadian regulation of transcription of genes encoding diverse photosynthetic enzymes in another chilling-sensitive plant, tomato (Martino-Catt and Ort 1992). However, one cannot directly transpose those results to maize as it has been shown at the enzymatic activity level that the responses of maize (Kingston-Smith et al. 1997) and tomato (Sassenrath et al. 1990) to low temperature are clearly different. The impact of cold on the circadian clock in *A. thaliana* is also well known (Mikkelsen and Thomashow 2009). However, thale cress is a cold-resistant, freezing-tolerant plant while maize is cold-sensitive. Additionally, the circadian clocks of maize and *A. thaliana* may differ (Jończyk et al. 2011). Thus a detailed study on the effect of cold-treatment on the circadian regulation of the transcriptome in maize is necessary to solve the apparent discrepancy.

In parallel to the repression of genes related to the chloroplast, induction of expression of many genes related to carbohydrate metabolism was observed, including genes encoding crucial components of the primary carbon assimilation (PCA) pathway, i.e., carbonic anhydrase, phosphoenolpyruvate carboxylase, phosphoenolpyruvate carboxykinase, and RuBiSCO large-subunit-binding protein. In contrast, several genes related to starch synthesis were repressed, while a gene encoding sucrose phosphate synthase was induced weakly. Thus, the common response of both lines to the cold at the transcript level comprises, on the one hand, an inhibition of the light reaction of photosynthesis without distinct changes in the antioxidant defense, and—on the other—an up-regulation of the dark reaction of photosynthesis with a possible shift from starch synthesis to sucrose synthesis. This conclusion, however, is contradictory to the well-known decrease of the activity of many enzymes of the dark phase of photosynthesis in maize at low temperature (Foyer et al. 2002; Leipner and Stamp 2009). This discrepancy could be caused by the lower enzyme activity per se at low temperature, their higher degradation rate or other modes of an overriding post-transcriptional regulation of the amount and activity of the dark-reaction enzymes. Alternatively, the induction of transcription of the respective genes could represent a compensatory response to the depletion of the enzyme activity at low temperature.

Photosynthesis is the natural focus of interest of studies concerning the response of maize to low temperature since it is widely assumed as the main target of cold in that species (Foyer et al. 2002). It is, however, evident from our data that even the short cold treatment results also in profound changes all over the transcriptome. An analysis using the gene ontology system indicated several processes specifically induced or repressed at the transcript level by objectively identifying over-representation of genes from a given GO category among those responsive to the treatment. Such an analysis supported by statistical tools gives more solid information than plain listing of raw numbers of genes from a given functional category, as found in some earlier microarray studies. For that reason the GO system or its more compact relative GO slim are becoming the standard tool for microarray data interpretation (Filichkin et al. 2011; Khan et al. 2010).

Basing on the global GO analysis we propose that, in addition to the previously discussed chloroplast functioning and carbohydrate metabolism, several other biological functions are affected by cold. One such response involves the cell wall structure and properties (over-represented GO category: cell wall organization or biogenesis). A similar response, albeit with less profound expression changes and concerning a lower number of genes, was observed earlier in maize subjected to moderate chilling (Trzcinska-Danielewicz et al. 2009). This much stronger expression of the trait in maize treated with severe cold additionally points to the cell wall as an important target of low temperature in maize. As far as we know, no studies have been published on the cell wall properties in cold-treated maize, therefore it is not known whether the low temperature directly affects the cell wall structure, like it does in dicots (Solecka et al. 2008), or whether the cell wall act as a receptor of the low temperature stress in maize. A role of the cell wall in the signal transduction to protoplasts has been suggested earlier (Fleming 2005). We discuss this question further in the section on differential gene response.

Another response to the cold common to the two lines was a widespread modulation of gene expression, evidenced not only by the substantial number of genes affected, but also by the over-representation of genes related to regulation of transcription (GO categories: regulation of gene expression; transcription, DNA dependent). Since at the same time the GO category “protein phosphorylation” was over-represented, a modulation of both the amount and activity of numerous proteins can be expected, suggesting an induction of acclimation of both lines to the cold.

The conclusion of a deep reconstruction of the leaf functioning is further confirmed by the observation that as many as ca. 250 genes (i.e., 10 % of all the genes responding) encoding stress-related proteins, transcription factors and proteins involved in hormone signaling/metabolism demonstrated a change in expression upon cold treatment, mostly induction. Although the stress-related genes were mostly those known to respond to drought stress, several others were also found related to wounding and pathogen attack, i.e., possibly functionally linked to the apoplast. This again underlines the potential role of the cell wall in low-temperature stress perception. Among the transcription factors affected by cold, members of the WRKY superfamily and the R2R3-MYB family were particularly numerous. In *A. thaliana*, WRKY proteins control, among other processes, pathogen defense and senescence (Eulgem et al. 2000) while R2R3-type MYB transcription factors are involved in the regulation of secondary metabolism and developmental processes (Stracke et al. 2001). Apart from the transcription factors from these two groups many others related to developmental processes were found among those changing expression at low temperature in both lines, e.g., those related to the metabolism of plant hormones or hormone signal transduction. These results confirm the suggestions of others that the response of maize to cold treatment involves the action of the growth inhibitors ABA and ethylene (Janowiak and Dörffling 1996; Janowiak et al. 2002; Szalai et al. 2000). However, we also found pronounced changes of expression of genes related to the growth stimulators auxins and cytokinins. Among the auxin-related genes was *TIR1*, repressed at low temperature in both lines. In the presence of auxin the TIR1 protein interacts with Aux/IAA transcriptional repressor proteins and mediates their degradation (Mockaitis and Estelle 2008), thereby allowing expression of auxin-responsive genes. Repression of *TIR1* suggests, therefore, a down-regulation of those genes. On the other hand, we also observed induction of orthologs of several *A. thaliana* auxin response factors (ARF1, ARF2, ARF6, ARF12, ARF16). These contrasting effects related to auxin were unlikely to be related to senescence induction, which could be expected as a response to the severe cold stress (Kratsch and Wise 2000), since no known auxin-induced senescence genes changed their expression except for *SAG12* and *SERK1* demonstrating repression and weak induction, respectively.

Among the cytokinin-related genes, several ones encoding cytokinin oxidase/dehydrogenase were repressed. The enzyme is a negative regulator of cytokinin level responsible for hormone homeostasis (Schmülling et al. 2003). Additionally, a gene encoding cytokinin-*N*-glucosyltransferase 1, responsible for irreversible inactivation of cytokinins (Sakakibara 2006), showed repression and another one, encoding cytokinin-*O*-glucosyltransferase 2, responsible for reversible inactivation of cytokinins (Martin et al. 1999; Sakakibara 2006), was induced. One may thus expect an increased cytokinin level in the leaf. An increased cytokinin supply from the root in cold-treated maize was suggested earlier (Sowiński et al. 1998). Altogether, these observations strongly suggest that diverse developmental processes are modulated in maize leaves by severe cold, not only those dependent on ABA and ethylene.

### Differential response

We have identified 66 genes showing different response to the cold stress in the two inbred lines studied. All these genes are excellent candidate causative agents of the divergent cold-tolerance of the ETH-DH7 and ETH-DL3 lines and possibly of other maize lines as well.

Ten plastid-related genes were among the 66 showing a differential pattern of response to cold-treatment. None of them could be directly connected to the functioning of the photosynthetic apparatus; they were rather involved in the general functioning of the chloroplast, including its development. This could reflect the fact that the lines used in our experiments had originally been selected on the basis of the efficiency of their photosynthetic apparatus developed at low temperature (Fracheboud et al. 2004) and is also in line with the idea that the development of the photosynthetic apparatus is the main target of cold stress (Nie et al. 1995; Kutík et al. 2004; Sowiński et al. 2005). However, most of those plastid-related genes demonstrated a more-positive cold-induced expression change in the cold-sensitive ETH-DL3 line than in the cold-tolerant ETH-DH7. One of those genes, GRMZM2G701020, showed one of the strongest expression changes among all the genes responding to the cold, an almost 24-fold induction in the ETH-DL3 line, with almost no change in ETH-DH7. According to the InterPro domain description (IPR021137), it encodes the L35 protein of the chloroplast ribosome. This could reflect rebuilding of the ETH-DL3 chloroplasts in response to the cold. Likewise, the expression of lipoxygenase 11 was up-regulated much more in ETH-DL3 than in ETH-DH7. This gene is an ortholog of *AtLox2* encoding lipoxygenase 2 from *A. thaliana* reported to be induced in response to wounding and drought (Bell and Mullet 1991; Bell et al. 1995). Since lipoxygenases are involved in the biosynthesis of stress hormones—jasmonates and abscisic acid (Creelman et al. 1992; Vick and Zimmerman 1987), one could suggest that the cold stress affected the ETH-DL3 seedlings more strongly than it did the ETH-DH7 ones.

Beside the plastid-related genes, profound expression changes differentiating the two lines studied were found for several genes encoding proteins related to other cell compartments: mitochondria, endoplasmic reticulum, Golgi apparatus, nucleus, vacuole and tonoplast, plasma membrane and cell wall, as illustrated schematically in Fig. 3. However, the number of those genes is too small to allow any statistically-supported analysis.

Perhaps the most striking difference between the responses of the two lines to the cold is the induction of expression of several genes encoding membrane proteins in the cold-tolerant ETH-DH7 line as opposed to a lack of changes or even a repression of those genes in the cold-sensitive ETH-DL3 line. Membranes are well known as a target of low temperature in maize (for review see: Greaves. 1996; Marocco et al. 2005; Leipner and Stamp 2009). This work adds to the already known picture, as it is tempting to infer that transport through the cell membranes is up-regulated in response to the low temperature uniquely in the cold-tolerant line. This up-regulation apparently concerned the tonoplast as well, as suggested by the ETH-DH7-specific induction of expression of genes encoding vacuolar ATP synthase subunit E (GRMZM2G078839) and an ortholog of a rice putative cation efflux family protein (GRMZM5G862882) of predicted vacuolar localization.

The membrane-related genes showing differential response to low temperature are involved not only in transport, but also in signal transduction. A role of membranes in cold perception has been postulated before (Murata and Los 1997). Results of our work show, however, some new aspects of the problem. One of the genes involved in signal transduction, GRMZM2G028568 encoding a homolog of rice cell wall-associated receptor-like cytoplasmic kinase OsWAK10d, is of particular interest since it is one of the kinases that link functionally the plasmalemma with the cell wall and could be involved in the relaying of signals from the cell wall inwards (Kanneganti and Gupta 2008). As is evident from both the detailed and global analyses of genes showing similar responses to the low temperature in both lines, the genes encoding proteins related to the cell wall form a prominent group, suggesting that the cell wall is a crucial target of the low temperature action. The cold-tolerant ETH-DH7 line seems to respond to the cold-induced cell wall changes, perhaps sensed as an altered mechanical tension, by enhancing the capacity to relay the information about such changes to the cell interior. A more efficient signal transduction pathway could speed up and/or enhance compensatory cell reactions to the adverse effects of the low temperature.

**Fig. 4:**
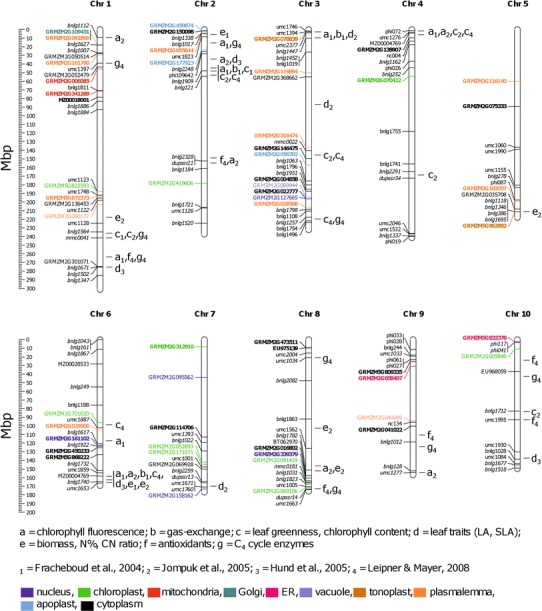
Chromosomal localization of genes responding differentially to cold treatment in the two maize lines and of QTLs associated with morpho-physiological traits expressed under low temperature conditions. On a physical map of maize chromosomes marked are positions of genes showing statistically significant (*p* < 0.1) interaction of line and temperature effects; molecular markers used to construct the ETH-DH7 × ETH-DL3 genetic map (Fracheboud et al. 2004); and QTLs found by Fracheboud et al. (2004), Hund et al. (2005), Jompuk et al. (2005), and Leipner and Mayer (2008). Coordinates of genes represented by microarray probes are given according to the Maize Genome Sequencing Project, and of molecular markers according to the MaizeGDB. Markers of known or estimated localization are shown in normal or italic typeface, respectively. *Bold* typeface indicates more-positive or less-negative expression change of a gene in response to cold in ETH-DH7 than in ETH-DL3. Detailed data on proteins identified by ensembl gene ID are given in Table S3. *Different colors* indicate the predicted cellular localization of products of genes responding differentially to cold treatment following the color-code at the *bottom* of the figure

Our transcriptomic study was performed on the inbred lines previously used for QTL mapping of cold sensitivity (Fracheboud et al. 2004; Hund et al. 2005; Jompuk et al. 2005; Leipner and Mayer 2008), which allowed us to attempt matching the results of those earlier studies with the present analysis. To this end we marked on a physical map of the maize genome the localization of the SSR markers used previously to generate the ETH-DH7 × ETH-DL3 genetic map (Fracheboud et al. 2004), and aligned by linear regression the previously published QTLs on the physical map (Fig. 4).

Among the 66 differentially responding genes, many overlapped or were very close to QTL regions associated with certain biometrical, physiological, or biochemical parameters related to the cold-performance of the lines studied (Fig. 4). Below we discuss the genes found next to the major QTLs. An ortholog of the *Triticum urartu* cell cycle control gene *CWF19*-*like 2* (GRMZM2G301071) at about 270 Mb on chromosome 1 is associated with QTLs for leaf traits (Hund et al. 2005), chlorophyll fluorescence (Fracheboud et al. 2004), as well as levels of antioxidants and C_4_ cycle enzymes (Leipner and Mayer 2008) at 15 °C. Near the telomere of chromosome 3, GRMZM2G078839 encoding vacuolar ATP synthase subunit E is located closely to a QTL associated with chlorophyll fluorescence, gas exchange and leaf traits. On the same chromosome at about 150 Mb lies a QTL associated with the chlorophyll content under low temperature conditions and, linked even more tightly, with leaf growth and greenness under optimal temperature (Fracheboud et al. 2004; Jompuk et al. 2005). Close to this QTL we identified GRMZM2G316474 coding for a putative leucine-rich repeat receptor-like protein kinase and GRMZM2G146475 encoding glutathione *S*-transferase GST28. On chromosome 5 at ca. 211.5 Mb, next to a QTL related to biomass accumulation under cold field conditions, lies GRMZM5G862882 coding for a putative cation efflux family protein, and on the short arm of chromosome 2 GRMZM2G177923 encoding glycoside hydrolase lies near a QTL for chlorophyll fluorescence, leaf traits, and C_4_ cycle enzymes. Near the telomere of the short arm of chromosome 4, GRMZM2G138907 encoding GDP-mannose 3,5-epimerase 2 neighbors a QTL associated with chlorophyll fluorescence and leaf greenness. Finally, on chromosome 6 at ca. 158 Mb a sequence represented by probe MZ00004769 coincides with the major QTL which alone explains a large part of the variation of photosynthesis at low temperature (Leipner 2009).

Among the above genes, four seem to be of particular interest because of their putative functions as inferred from their GO annotations in the class Biological Process. One is GRMZM2G301071, whose wheat ortholog has an annotation “Response to freezing” (Source: EnsemblPlants/Gramene). GRMZM2G078839 has annotations “ATP hydrolysis coupled proton transport”, “Response to cold”, “Response to salt stress”, “Golgi organization” and “Plant-type cell wall biogenesis” (UniProtKB/TrEMBL: B4FB71_MAIZE). The third one, GRMZM2G146475 has GO annotations “Translation” and “Response to stimulus” (Panther Classification System). The fourth one is GRMZM2G138907 having the annotation “l-ascorbic acid-biosynthesis process” (EnsemblPlants/Gramene). For two of these genes (GRMZM2G30107 and GRMZM2G078839) their annotations indicate a direct link to low temperature treatment and for another two (GRMZM2G146475 and GRMZM2G138907) an indirect one, as they are possibly involved in redox homeostasis. The most interesting is, however, the transcript hybridizing to probe MZ00004769. This probe apparently represents an intronic sequence of the gene GRMZM2G074906, identified as a putative transposable element. The same sequence is found in one more genome region at ca. 4 Mb on chromosome 4. No genes are ascribed to this region but, remarkably, four QTLs are localized there. The corresponding transcript is very strongly induced (ca. 16-fold) by the cold in the cold- sensitive ETH-DL3 line, showing no response in the cold-tolerant line. This behavior and the co-localization with several QTLs, including the major one on chromosome 6, warrant a closer scrutiny of these loci; one is tempted to speculate that the intronic and, apparently, non-genic sequences harbor a regulatory RNA(s).

To check if the discussed co-localization of genes and QTLs is not merely coincidental, we performed mathematical modeling of the case. Shortly, all microarray probes were mapped on the maize genome and the 66 probes showing differential response were checked for random localization near QTLs shown on Fig. 4 (a complete description of the model and detailed results are available on request). Among those probes, two (of the five discussed above) were found to be linked with a QTL with p < 0.10: MZ00025856 (representing GRMZM2G078839 located near chromosome 3 telomere) and MZ00004769 (representing an intronic sequence of the gene GRMZM2G074906 on chromosome 6). Several other probes were also found linked with QTLs with a lower statistical significance (0.10 < *p*<0.20). These modeling results should be treated with care because of the rather large uncertainty of QTLs localization, but they constitute yet another argument for the importance of the genes in question.

Our study was not designed to identify with certainty individual genes responsible for the divergent behavior at low temperature of the two inbred lines selected for contrasting cold-tolerance of photosynthesis. However, the close association of some differently regulated cold-responsive expressed regions with major QTLs for cold-performance indicates the direction of future studies. The obtained results also clearly point to the cell membrane/cell wall deserving scrutiny as potential sensors of low temperature and/or primary effectors modulating diverse aspects of the cold response. The same concerns the cold-induced disturbance of the circadian rhythm as a reason for the repression of genes related to the light reaction of photosynthesis and modulation of other clock-sensitive genes representing a substantial fraction of the maize transcriptome (Hayes et al. 2010; Khan et al. 2010; Jończyk et al. 2011).

## Electronic supplementary material

Below is the link to the electronic supplementary material.

Supplementary material 1 (DOC 52 kb)

Supplementary material 2 (XLS 692 kb)

Supplementary material 3 (XLS 70 kb)

Supplementary material 4 (XLS 47 kb)

Supplementary material 5 (XLS 152 kb)

Supplementary material 6 (JPEG 3227 kb)

Supplementary material 7 (JPEG 2796 kb)

Supplementary material 8 (JPEG 3132 kb)

Supplementary material 9 (JPEG 4372 kb)

Supplementary material 10 (JPEG 3009 kb)

Supplementary material 11 (JPEG 2810 kb)

Supplementary material 12 (XLSX 14 kb)
