# Supplementary material for: Genome-wide transcriptomic analysis of response to low temperature reveals candidate genes determining divergent cold-sensitivity of maize inbred lines
Source: Plant Mol Biol. 2014 Mar 13;85(3):317–31. doi: 10.1007/s11103-014-0187-8 (PMC4018516; doi:10.1007/s11103-014-0187-8)
Supplement: Supplementary file 1 — Supplementary material 1 (DOC 52 kb) [file 11103_2014_187_MOESM1_ESM.doc]

Genome-wide transcriptomic analysis of response to low temperature reveals candidate genes determining divergent cold-sensitivity of maize inbred lines. Plant Molecular Biology, Sobkowiak A, Jończyk M., Jarochowska E., Biecek P., Trzcinska-Danielewicz J., Leipner J., Fronk J., Sowinski P., Faculty of Biology, University of Warsaw, pawes@biol.uw.edu.pl

Table S1. Primers for qRT-PCR

| ID | Expected product length [bp] | Forward primer (5’ ® 3’) | Reverse primer (5’ ® 3’) |
| --- | --- | --- | --- |
| MZ00057331 | 230 | TACGGCTCAAGTTCACCAAG | CTCACCAAACCTCCTCTCCATC |
| MZ00057095 | 110 | GGTTCGGTTCGCTTCGTTC | AGCCTTGGGTCTCTTGGTCC |
| MZ00055486 | 590 | CCTCGCTTTGCTTCTCGT | TATTCACTTTCACTCATTTCGGTA |
| MZ00044415 | 250 | GAGCCGAGAGTGAGACAACCA | TTCAGCGTGTAGAGGGACCA |
| MZ00043929 | 580 | GCCCTCCTCTGCCTCCTC | TGCCTTCCTCTCCTTCCTCTC |
| MZ00043811 | 135 | GCTTTTCTGCCTCCTTTTTACTGC | TCCTCTCCCTCCCCATTTACC |
| MZ00043252 | 190 | ACCGAGAGGGAGGGCAAG | TCAAAACCAAAGCGACACCA |
| MZ00043222 | 180 | CAAACCAAAGGCACAGGC | CGACATCACTCACAAGCATCA |
| MZ00033462 | 120 | GCTGTTGTTCCGAGGTTTTTAG | CGGTCATTTTATTTGTTTATTGTCC |
| MZ00033014 | 220 | GTCAAACCAGGACCCGATAAA | GCCGTCGCAGAAAACACAG |
| MZ00032477 | 270 | GGACTACAAGAAACGGCAGGAAA | GCAGAAGAGCAGGCAGATACATT |
| MZ00031651 | 260 | GGAAGGTGATGGGCAAGG | GCGGGAAGTAGAAGGGGTAG |
| MZ00031472 | 160 | AGCCAAAGACAAACAGGAGA | TACCAGCAACGCCAAAAGG |
| MZ00029953 | 190 | CGCTCTGTTTCCTGCTGTCTG | CTCCTCCGCCTCCATTCTG |
| MZ00026299 | 150 | GCACACCACACAACACAACACA | CACCACCACCACCAACAGG |
| MZ00025856 | 230 | ACGCTGGGCTCCTTTTCA | CGGTGGGTTTTGTTTGTCTC |
| MZ00024672 | 218 | GATACAAGGCGAGGGAGGTG | ATACAGAGTGAGAAGCGAACAAAA |
| MZ00024226 | 250 | CCTGGTTTCTTCATTGGCTTCTG | CCTTTCCTGCCCTTACCTCTC |
| MZ00023628 | 80 | CTTTTGCTTCCCTTCCTGCTACC | ATCCTGATGATTCTGTCGCCA |
| MZ00022886 | 240 | GGAAGGGAGAAAGAAGAAGGAA | TCGGAAAGGTAGAACGGGTAG |
| MZ00019152 | 170 | GTGGTGGATGACGCCCTACTC | CTGCTTGACGATGCGATTGC |
| MZ00018707 | 245 | TCCCATTTGTTCCTTTTCCTC | ATTCGCCCCTACTCCCATTA |
| MZ00016005 | 230 | TGCTGACCAACGCAACCAT | CACATCCCACACCAAACACACT |
| MZ00014938 | 190 | AGACGCTGACGGACAACACG | GCGAAGACCTGGAAGAACACC |
| MZ00014358 | 180 | CTTCGCCCCTTCCTCTCTC | AGGCACTTCTCGGGTTTAG |
| MZ00004986 | 230 | TTCTCTTGTTCTGTGTAGTGTGCTTTC | CGGTGTTTGTTTATTGCGTTCA |
| MZ00003980 | 225 | TTCCCATTACCTCTTGCCACTA | GGGCTACTGCTGCTTCCTG |
| MZ00001641 | 280 | TGAACTCGCCTGGTTTTCTGTG | ACTTGCTCGCTTTGGCTGTATC |
| MZ00001058 | 270 | CCACCCCACTCGCAACAA | TTATCCTTCCCCATCACACTTCTTA |
