# Supplementary figures and images for: Genome-wide transcriptomic analysis of response to low temperature reveals candidate genes determining divergent cold-sensitivity of maize inbred lines
Source: Plant Mol Biol. 2014 Mar 13;85(3):317–31. doi: 10.1007/s11103-014-0187-8 (PMC4018516; doi:10.1007/s11103-014-0187-8)

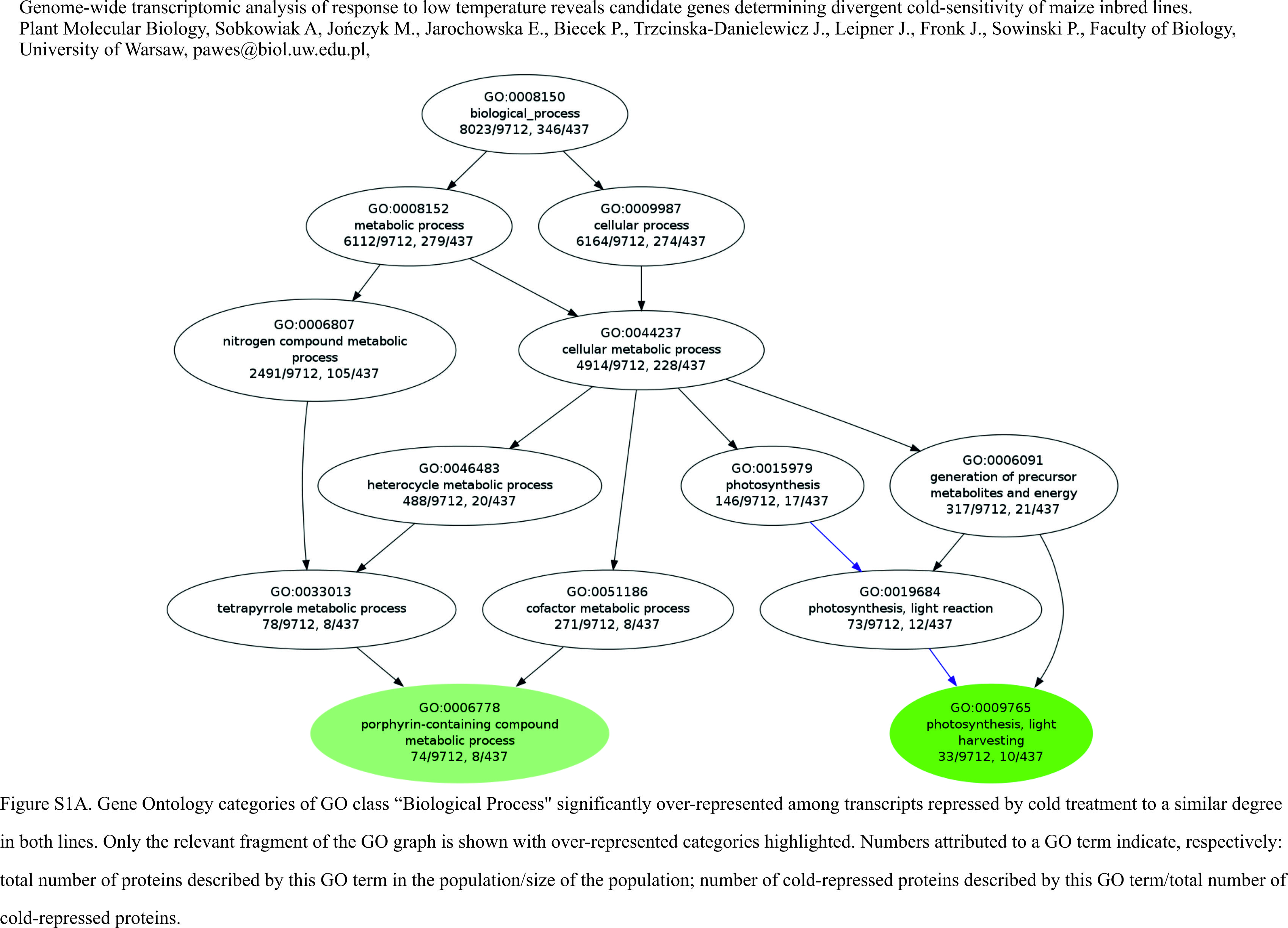

Supplement: Supplementary file 6 — Supplementary material 6 (JPEG 3227 kb) [file 11103_2014_187_MOESM6_ESM.jpg]

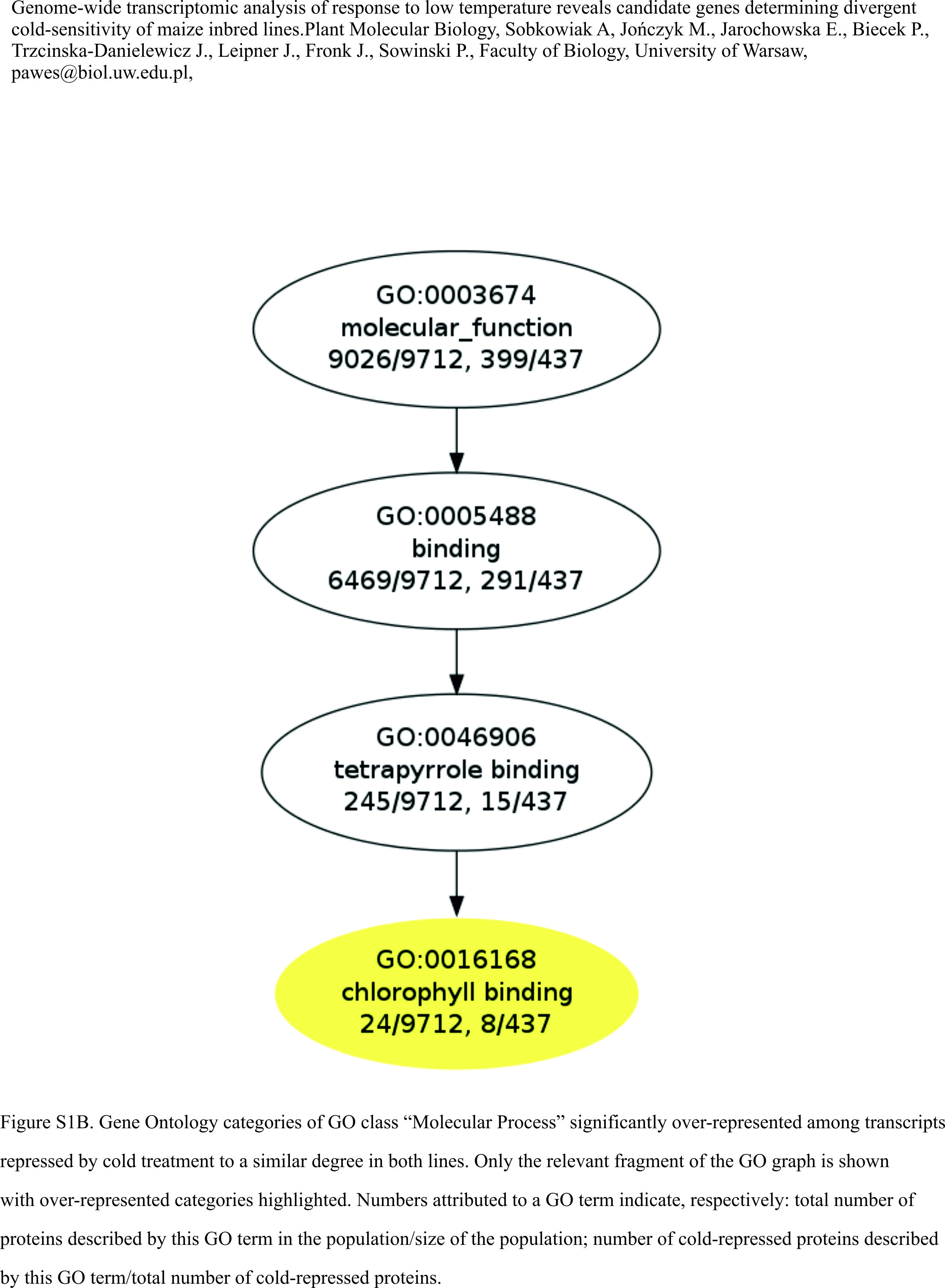

Supplement: Supplementary file 7 — Supplementary material 7 (JPEG 2796 kb) [file 11103_2014_187_MOESM7_ESM.jpg]

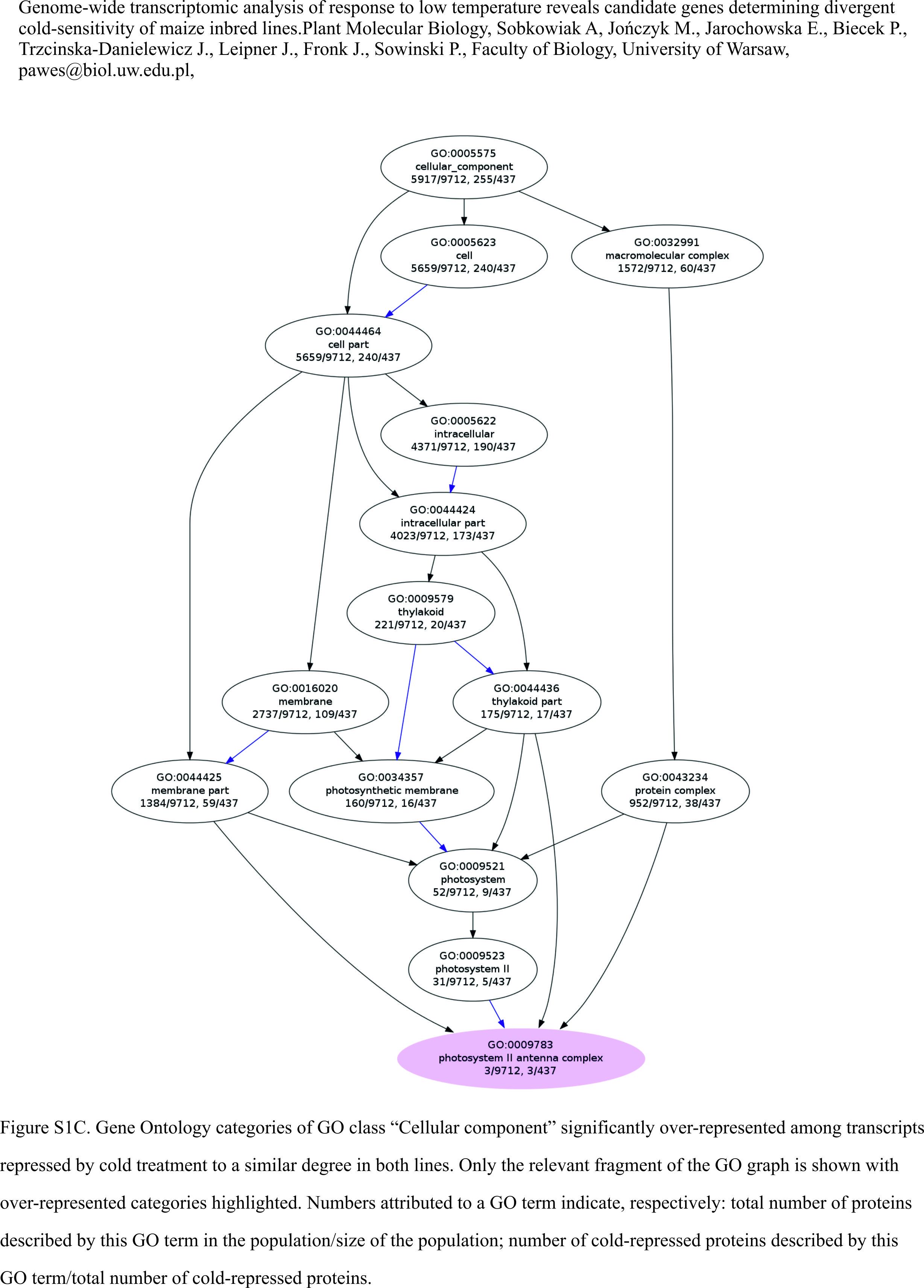

Supplement: Supplementary file 8 — Supplementary material 8 (JPEG 3132 kb) [file 11103_2014_187_MOESM8_ESM.jpg]

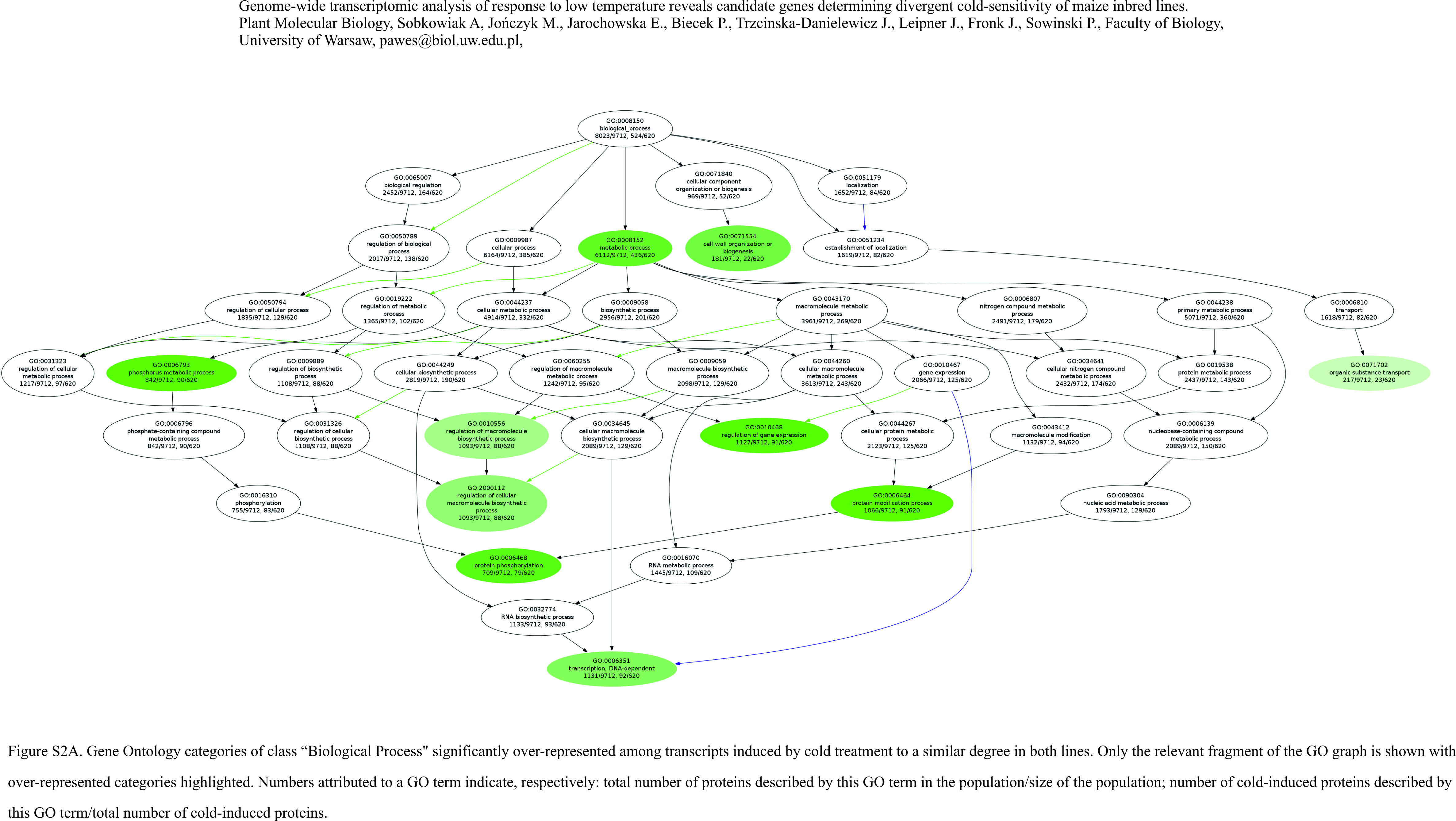

Supplement: Supplementary file 9 — Supplementary material 9 (JPEG 4372 kb) [file 11103_2014_187_MOESM9_ESM.jpg]

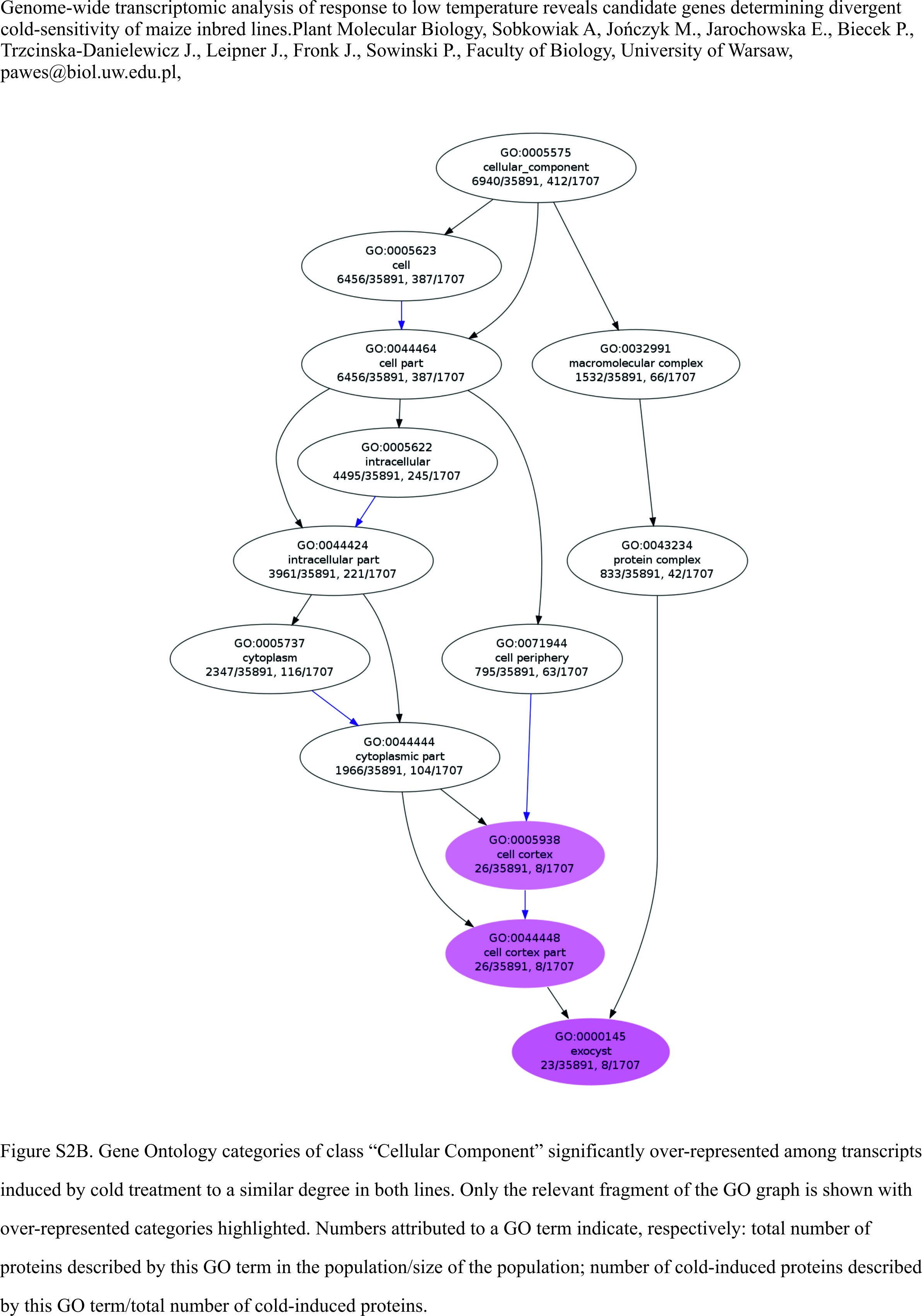

Supplement: Supplementary file 10 — Supplementary material 10 (JPEG 3009 kb) [file 11103_2014_187_MOESM10_ESM.jpg]

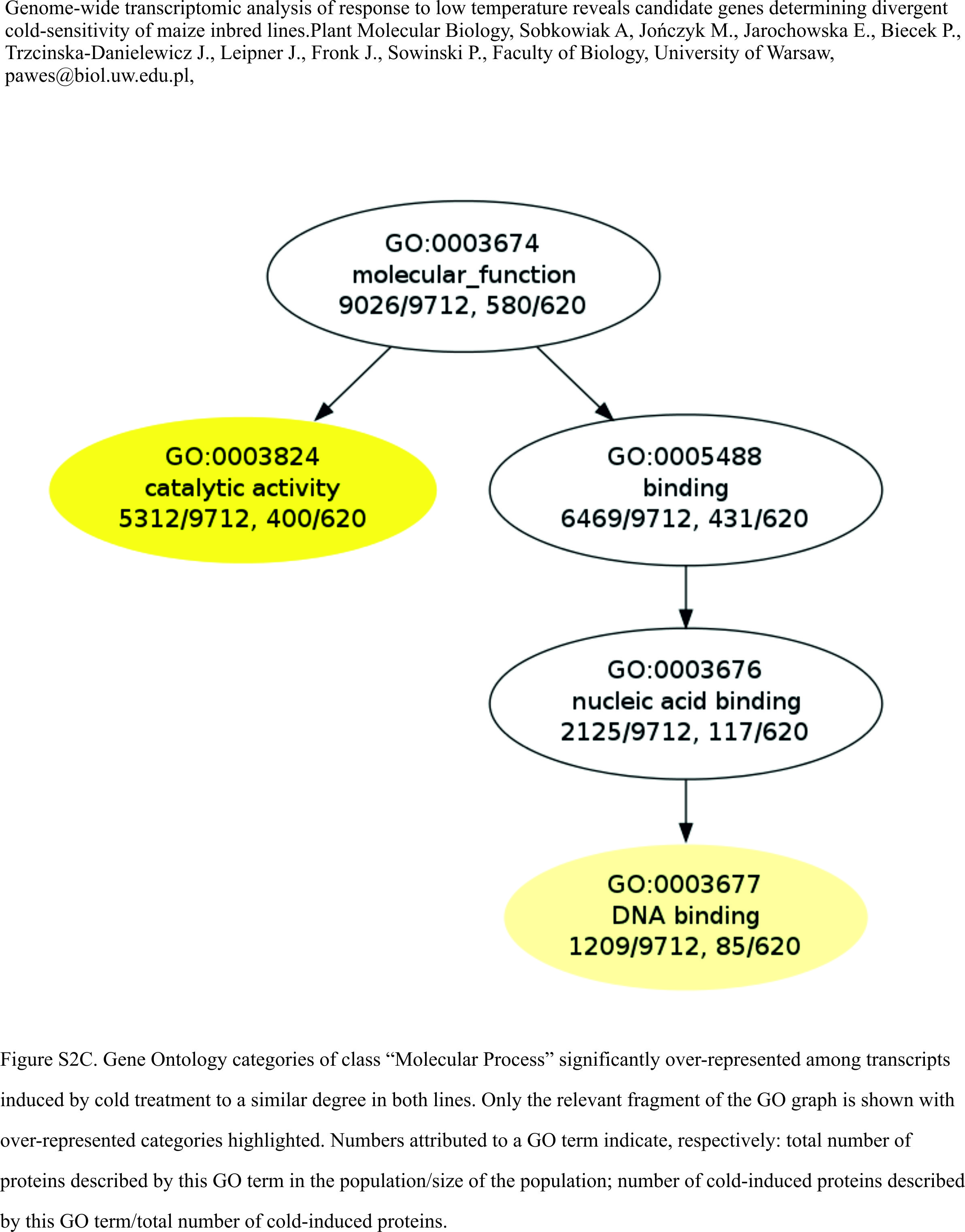

Supplement: Supplementary file 11 — Supplementary material 11 (JPEG 2810 kb) [file 11103_2014_187_MOESM11_ESM.jpg]
